# Comparative genomic analyses provide new insights into phylogenetic and functional diversification in genus *Fenollaria*

**DOI:** 10.3389/fmicb.2026.1862120

**Published:** 2026-08-04

**Authors:** Shaojing Wang, Lijuan Kang, Mei Li, Xiaodong Zhou, Bowen Li, Fei Wang, Jinhuan Meng, Changying Li, Kuo Yang

**Affiliations:** 1 Tianjin Institute of Urology, The Second Hospital of Tianjin Medical University, Tianjin, China; 2 Tianjin Key Laboratory of Precision Medicine for Sex Hormones and Diseases, The Second Hospital of Tianjin Medical University, Tianjin, China

**Keywords:** comparative genomics, *Fenollaria*, pan-genomes, phylogenomic analysis, urobiome

## Abstract

The genus *Fenollaria* has gained attention due to its associations with human prostate cancer, colorectal cancer and other diseases. The higher abundance of *Fenollaria* was believed to be associated with biochemical recurrence of prostate cancer but remission of colorectal cancer. Owing to the fastidious growth requirements of *Fenollaria* species in laboratory isolation and culture, the genomes of isolated strains is rarely available. Consequently, only limited comparative genomic studies have been conducted, leaving knowledge gap regarding the genomic diversity, distribution of functional genes, and evolutionary relationships, which hindered the understanding of ecological adaptation and mechanism exploration of *Fenollaria*. Here, a large-scaled genomic investigation of *Fenollaria* genus was performed using four high quality MAGs generated in this study and publicly available genomic data. The four MAGs were constructed from urine metagenome samples from bladder cancer patients, which were under conditions of oligotrophy and limited oxygen. Four mono-clades were revealed by phylogenomic analysis, representing for three previously described species (i.e., *F. massiliensis, F. timonensis*, and *F. sporofastidiosus*) as well as a novel proposed *Fenollaria* species. The divergences among these clades were also supported by genome-wide G + C content, ANI and AAI values. The functional difference between clades were revealed by the distribution of clade-specific genes in COG categories, as well as the biased distribution of ARGs, VFs, and CRISPR-Cas systems.

## 1 Introduction

The *Fenollaria* genus was designated decade ago (Pagnier et al., 2014), and had garnered considerable attention owing to its association with prostate cancer, colorectal cancer and several other diseases. Previously, Hurst et al. raised the anaerobic bacteria biomarker set (ABBS) as a prognostic biomarker linked to human prostate cancer (PCa) risk, which consisting of *Fenollaria* and other four bacteria genera. A significantly high rate of biochemical recurrence was observed in donors with at least one of the ABBS genera in the urinary tract (Hurst et al., 2022). Last year, Cai et al. suggested that *Fenollaria* was associated with remission of colorectal cancer (CRC). The *Fenollaria* were significantly suppressed in patients with CRC and its abundance in fecal microbiota had significant negative correlation between carcinoembryonic antigen (CEA) (Cai et al., 2025). The *Fenollaria* were also reported associated with other human diseases. The increased abundance of *Fenollaria* was associated with uric acid (UA) stones (Liu et al., 2025) and also observed in fecal samples from patients with major depressive disorder (MDD) (Prandovszky et al., 2025).

The taxonomic classification and genomic investigation of genus *Fenollaria* was raised in the past decade. Thus, far, this genus consisted of at least three anaerobic species, i.e., *F. massiliensis* (Pagnier et al., 2014), *F. timonensis* (Durand et al., 2017; Lo et al., 2020), and *F. sporofastidiosus* (Hurst et al., 2022). The *F. massiliensis* was originally isolated from an osteoarticular sample, with genome size of 1.71 Mb and G + C content of 34.46% (Pagnier et al., 2014). Subsequently, isolates of this species were identified from a variety of clinical specimens using the matrix-assisted laser desorption/ionization time-of-flight mass spectrometry (MALDI-TOF MS) strategy, including bone tissue, abscesses, various skin, and soft tissue infections and sperm samples (Boiten et al., 2018). However, their genomes were not determined. *F. timonensis*, the second species designated in *Fenollaria* genus, was originally identified from human gut microbiota. The *F. timonensis* species exhibited 16S rRNA gene sequence similarity of 97.4% to *F. massiliensis* (Durand et al., 2017; Lo et al., 2020), with genome size of 1.74 Mb and G + C content of 35.7%. The third species within the genus *Fenollaria* was also proposed based on 16S rRNA gene sequence similarity lower than 97% to the closest published species, and was designated as *F. sporofastidiosus* possessing a 1.6 Mb genome with G + C content of 36% (Hurst et al., 2022). The genomic data of *Fenollaria* was limited owing to its fastidious growth requirements, which made the isolation and culture of *Fenollaria* species in the lab particularly challenging. Although the *Fenollaria* has gained attention due to its association with human diseases, limited genomic data raised gaps of detailed description of genomic diversity, functional genes distribution and evolutionary relationships between species, which hinders the landscape and further mechanism exploration in *Fenollaria*.

Here, a large-scaled genomic investigation of *Fenollaria* genus was performed using MAGs generated in this study and publicly available genomic data, aiming to the phylogenetic and functional diversification. The four MAGs were constructed from urine metagenome samples from bladder cancer patients. Urine is characterized by its oligotrophic nature, limited oxygen availability and variable pH in bladder. The urinary microbiome commonly referred to urobiome (Wolfe and Brubaker, 2019) was gradually revealed owing to the development of advanced sequencing technology (Mueller et al., 2017; Price et al., 2016; Wolfe et al., 2012). The urobiome had garnered attentions owing to its potential implications for human health (Govender et al., 2019; Thomas-White et al., 2018; Bucevic Popovic et al., 2018).

## 2 Materials and methods

### 2.1 Participant recruitment and sample collection

Urine samples of bladder cancer patients were collected at The Second Hospital of Tianjin Medical University, Tianjin, China. The ethical approval was obtained from the Ethics committee of The Second Hospital of Tianjin Medical University in December 2025 (KY2025K474). Written informed consents were obtained from all participants. Midstream urine samples from participants were obtained using sterile containers. Samples were transported to the laboratory on ice within 2 h and centrifuged at 12,000*g* for 10 min at 4 °C to collect microbial biomass. The pellets were frozen in liquid nitrogen, and subsequently stored at −80 °C.

### 2.2 DNA extraction and metagenomic sequencing

Genomic DNA was extracted from urinary microbial pellets using the U.CAD Nucleic Acid Extraction Kit (Suzhou Hongyuan Biotechnology Co., Ltd.) according to the manufacturer's instructions with quantity determined by NanoDrop^TM^ 2000. Sequencing libraries were prepared using the U.CAD Library Construction Kit (Suzhou Hongyuan Biotechnology Co., Ltd., China) with quality assessed by Qubit and Qsep. Libraries meeting the quality criteria (yield ≥300 ng, main peak 180–1,800 bp, and no significant primer-dimer peaks) were selected for subsequent sequencing. Paired-end sequencing runs of 150 cycles were performed on the Illumina NextSeq 500 platform using the NextSeq 500 v2 kit.

### 2.3 Metagenomic data analysis

Raw sequencing reads were quality-filtered and adapter-trimmed using TrimGalore v0.6.01 (github.com/FelixKrueger/TrimGalore) with default parameters. Host-derived contaminating reads were removed using HoCoRT (Rumbavicius et al., 2023). Contigs were assembled and MAGs were reconstructed using the metaWRAP pipeline v1.3.2 (Uritskiy et al., 2018). The taxonomic lineages of these MAGs were assigned using the classification workflow in GTDB-Tk workflow v2.7.2 (Chaumeil et al., 2022) with the Genome Taxonomy Database (GTDB) release v232 (Parks et al., 2022). MAG quality was assessed using the “predict” workflow of CheckM2 v1.1.0 (Chklovski et al., 2023). Only MAGs with completeness ≥90% and contamination < 5% were considered to be of “high quality” and retained for downstream analyses (Jiang et al., 2022; Singleton et al., 2021).

These MAG sequences originally generated in this study have been deposited to the NCBI GenBank database under accession numbers JBXXYA000000000, JBXXYB000000000, JBXXYC000000000, and JBXXYD000000000.

### 2.4 Public resourced genomes

For comparative analysis, publicly available genome sequences of *Fenollaria* were retrieved from the National Center for Biotechnology Information (NCBI) website (www.ncbi.nlm.nih.gov) and gcMeta (Sun et al., 2026) (gcmeta.wdcm.org). The gcMeta 2025 integrated over 2.7 million MAGs from 104,266 samples spanning various biomes. These publicly available genomes were also evaluated using the “predict” workflow of CheckM2 v1.1.0 (Chklovski et al., 2023). Consistent with the aforementioned criteria, only genomes classified as “high quality” were retained for further analysis (Jiang et al., 2022; Singleton et al., 2021). The taxonomic lineages of all retained publicly available genomes were also re-assigned using the classification workflow in GTDB-Tk v2.7.2 (Chaumeil et al., 2022) with the Genome Taxonomy Database (GTDB) release v232 (Parks et al., 2022).

### 2.5 Phylogenomic analyses

The phylogenomic tree was reconstructed using bcgTree v1.2.1 (Ankenbrand and Keller, 2016) with default parameters. Briefly, the bcgTree is an automated pipeline for phylogenetic tree reconstruction based on protein sequences of 107 essential genes extracted from bacterial genomes, relying on genes predicted by Prodigal v2.6.3 (Hyatt et al., 2010). The essential genes were identified using HMMER v3.4 (Eddy, 1998, 2009), followed by multiple sequence alignment (MSA) generated by MUSCLE v3.8.31 (Edgar, 2004). The bcgTree pipeline employed Gblocks v0.91b (Castresana, 2000) to remove unreliable regions from MSA prior to phylogenetic tree reconstruction. A maximum likelihood (ML) phylogenetic tree was then inferred using RAxML-NG v1.2.2 (Kozlov et al., 2019), with 1,000 rapid bootstrap replicates under the best-fitting substitution model “LG + I + G4” determined by ModelTest-NG v0.1.7 (Darriba et al., 2020). The ML tree was midpoint-rooted and visualized using iTOL (Letunic and Bork, 2021).

### 2.6 Comparative genomic analysis

Genome-wide protein-coding genes were predicted using Prokka v1.13 (Seemann, 2014). The pan-genome and the gene presence/absence matrix were generated using Roary v3.11.2 (Page et al., 2015) with GFF3 files as input and minimum BLASTP sequence identity threshold of 80%. Genes present in all genomes were defined as the hard-core genome, whereas those present in 90% ≤ *x* < 100% of genomes formed the soft-core genome. The shell-genes and cloud-genes were defined using thresholds of “15% ≤ *x* < 90%” and “*x* < 15%,” respectively. The Heaps' law, as proposed by Tettelin et al. (2008) was used to evaluate the openness of the *Fenollaria* pan-genome as previously described (Park et al., 2019). Briefly, a positive exponent (γ > 0) indicates an open pan-genome, whereas a negative exponent (γ < 0) indicates a closed pan-genome. Clade-specific genes were identified using Scoary v1.6.16 (Roder et al., 2024) based on the gene presence/absence matrix, with Benjamini–Hochberg adjusted *P*-value threshold of 0.05, together with sensitivity > 0.95 and specificity > 0.95.

Pairwise average nucleotide identity (ANI) values were calculated for all genomes using fastANI v1.3 (Jain et al., 2018) with the parameter “–fragLen 100,” which splits the query genome into fragments of 100 base pairs to improve the sensitivity of ANI estimation. Average amino acid identity (AAI) values were calculated using EzAAI (Kim et al., 2021). Pairwise ANI and AAI matrices were visualized using the ggplot2 package in R v4.4.1 (www.r-project.org).

### 2.7 Functional analyses

Representative protein sequences of the pan-genome were functionally annotated using eggNOG-mapper v2.1.13 (Cantalapiedra et al., 2021) against the eggNOG database v5.0.2. Orthologous assignments were performed using MMseqs2 v16.747c6 (Steinegger and Soding, 2017) with parameters including E-value threshold of 1e-10 and minimum alignment score of 50. The COG functional categories and KEGG Orthology (KO) were retrieved from the best-matched orthologs for further analysis. The antibiotic resistance genes (ARGs) were identified using RGI v6.0.8 with the CARD v4.0.1 database following the recommended pipeline (Alcock et al., 2023). Only ARGs classified as “Perfect” or “Strict” were retained for further analyses. Virulence factors (VFs) were identified in each genome using the -draft workflow of the MetaVF toolkit (Dong et al., 2024). A sequence was considered a VF-associated gene homolog when it exhibited >70% amino acid identity and >70% coverage against a reference gene in VFDB 2.0. The machine learning model CRISPRcasIdentifier (Padilha et al., 2020) was employed for CRISPR-Cas systems identification and classification.

## 3 Results

### 3.1 Phylogenomic analysis

Four high-quality MAGs were successfully reconstructed from bladder cancer urine samples in this study. The genome sizes ranged from 1.45 to 1.91Mb, with G + C contents ranging from 33.61% to 34.53%. These MAGs contained 1,332–1,799 protein-coding genes and no 16S rRNA genes were recovered. Based on taxonomic classification, three MAGs were assigned to *F. massiliensis*, whereas one MAG was classified as *Fenollaria* sp. (Supplementary Table S1). To determine the phylogenetic placement of these four urine-derived MAGs and to investigate the genomic diversity in this genus, publicly available *Fenollaria* genomes were also retained.

The dataset of *Fenollaria* genomes was constructed for comparative genomic analysis. This dataset consisted of 175 genomes, including 4 MAGs newly generated this study, five isolate genomes and 2 MAGs retrieved from NCBI genome database and 164 MAGs retrieved from gcMeta (Supplementary Table S1), with all taxonomy lineages classified using GTDB-tk. All genomes retained in this study exhibited high assembly quality, with an average completeness of 97.91% ± 3.04% and an average contamination rate of 0.70% ± 1.05%. The average contigs number for the 168 MAGs was 52, with an average N50 of 75.20kb. Although 16S rRNA gene sequence similarity serves as a classic indicators for phylogenetic analysis (Kim et al., 2014), only two nearly complete 16S rRNA gene sequences were recovered from these MAGs (i.e., GCMeta_00237611 and GCMeta_00237627), which were insufficient for phylogenetic inference.

**Figure 1:**
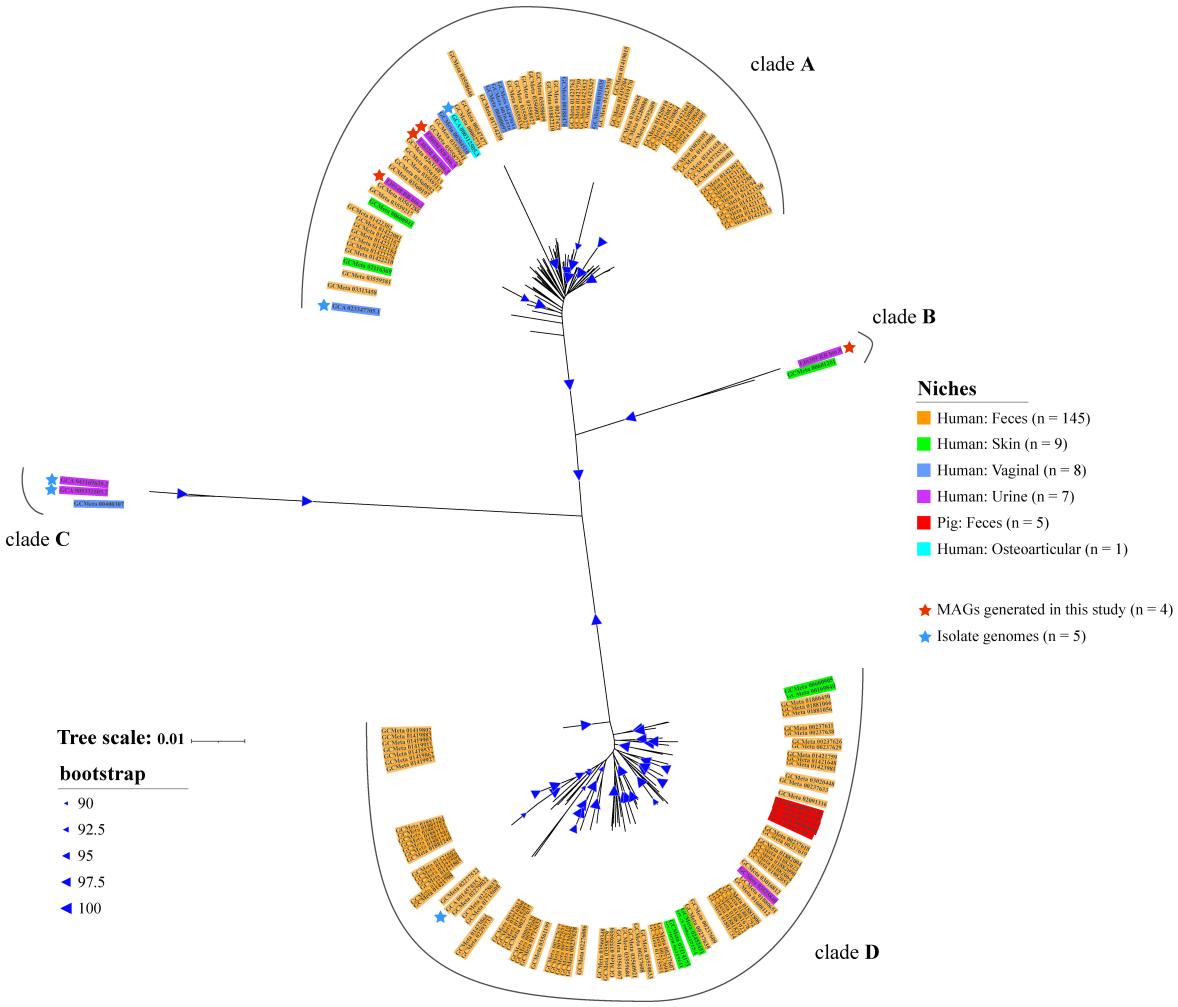
Phylogenomic tree of genus *Fenollaria*. Maximum likelihood phylogenomic tree was reconstructed based on 107 conserved single-copy protein coding genes. The tree was supported by 1,000 bootstrap replicates and unrooted.

**Figure 2:**
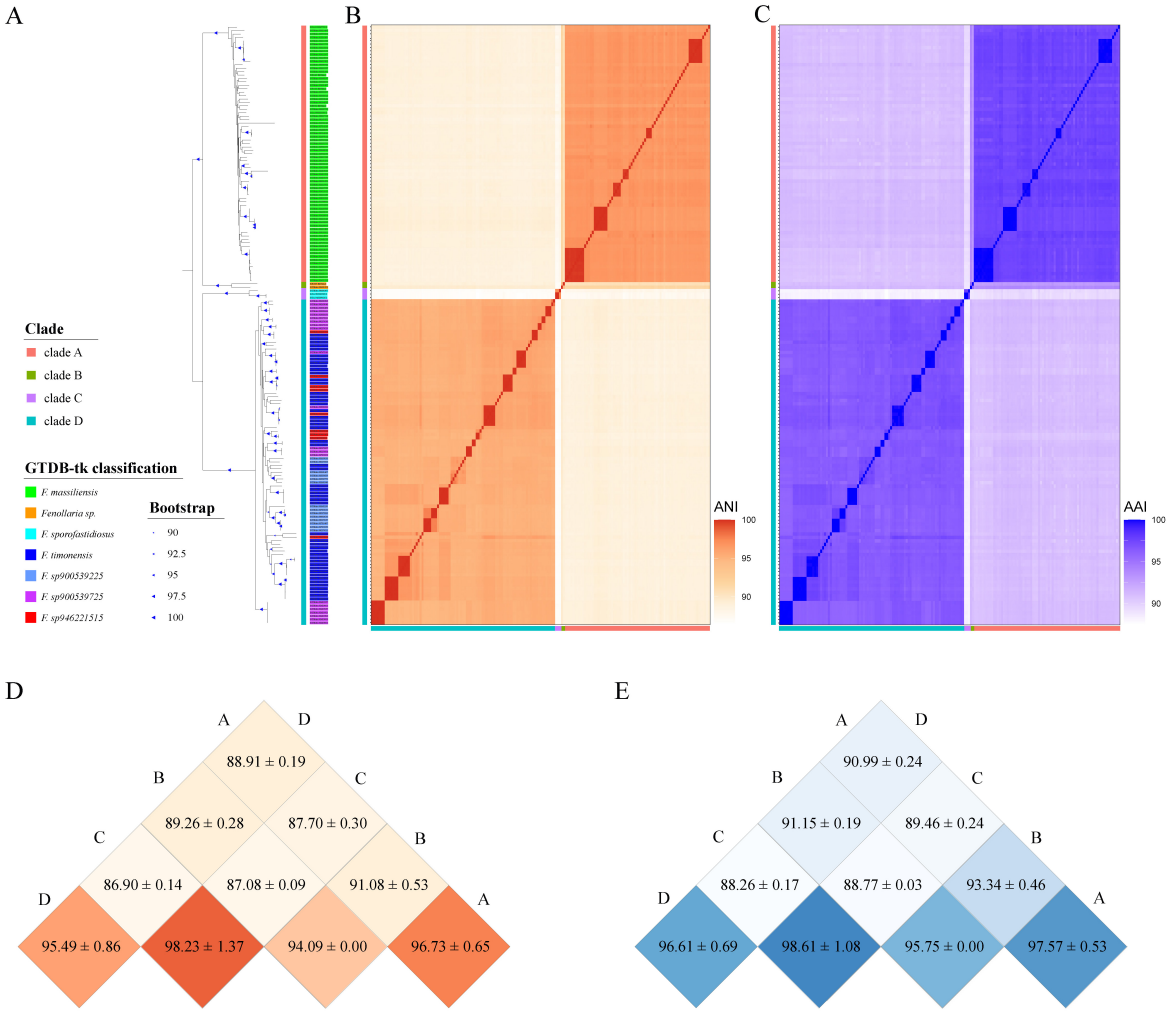
Comparative genomic analyses of *Fenollaria*. **(A)** Phylogenomic tree of genus *Fenollaria* with leaf labels marked by taxonomic lineages. **(B)** Distribution of pairwise genomic ANI values along with phylogenomic tree. **(C)** Comparison of ANI values inter and intra clades. **(D)** Intra- and inter-clade ANI values. **(E)** Intra- and inter-clade AAI values.

To investigate the phylogenetic relationships among *Fenollaria* species, a phylogenomic tree was reconstructed based on conserved single-copy protein coding genes recovered from genome datasets. Four monophyletic clades (i.e., clades A, B, C, and D) were identified (Figure 1). Clade A and D were the largest groups. Clade A comprised 75 genomes, including two isolate genomes obtained from human osteoarticular and vaginal samples, while the remaining 73 MAGs were derived from fecal (*n* = 62), vaginal (*n* = 6), urinary (*n* = 3) or skin (*n* = 2) samples. All genomes in clade A were classified as *F. massiliensis* by GTDB-tk, suggested a broad ecological adaptability of this species within the human host. Clade D consisted of 95 genomes, including one isolate genome obtained from human fecal sample and 94 MAGs derived from human feces (*n* = 82), skin (*n* = 6), and urine (*n* = 1) samples, as well as pig fecal samples (*n* = 5). The taxonomic classification of genomes within clade D was inconclusive. Although GTDB-tk assigned these genomes to at least four species, none of the species formed a mono-subclade within clade D (Figure 2A). Briefly, 51 genomes were assigned to *F. timonensis*, whereas 21 MAGs were classified as *Fenollaria* sp900539725, 14 MAGs as *Fenollaria* sp900539225 and 9 MAGs as *Fenollaria* sp946221515.

In contrast, clade B and C were relatively small. Clade B consisted of two MAGs recovered from human urine and skin samples, both of which remained unclassified at the species level. The Clade C consisted of three genomes, including two isolate genomes and one MAG derived from human urine and vaginal samples. All these genomes represented for *F. sporofastidiosus*. Notably, close phylogenetic relationship of pig feces-derived MAGs was inferred for their neighboring location within Clade D. No biased distribution of urine-derived genomes was observed, as these MAGs were distributed across all of the four clades. In contrast, vaginal-derived genomes were observed only within Clades A and C, suggested potential niche-specific phylogenetic distribution.

The cladistic relationships and genomic diversity were further evaluated based on the biased distribution of ANI and AAI values, both of which are robust measurements of genomic relatedness between strains (Figure 2). The biased distribution of ANI values inferred in heatmap was consistent with the topology of the phylogenomic tree, with higher ANI values observed within clades and lower values between clades (Figure 2B). The similar pattern was also observed for AAI values (Figure 2C). Notably, the heatmap distributions of ANI and AAI further suggested considerable genetic diversity within individual clades. The average intra-clade ANI values of genomes in clades A, B, C, and D were 96.73%, 94.09%, 98.23%, and 95.49%, respectively, whereas the average inter-clade ANI values ranged from 86.90% to 91.08% (Figure 2D). Similarly, the average intra-clade AAI values of genomes in clades A, B, C, and D were 97.57%, 95.75%, 98.61%, and 96.61%, respectively, while the average inter-clade AAI values ranged from 88.26% to 93.34% (Figure 2E). It is generally recognized that a 16S rDNA sequence similarity of 98.65% serves as the threshold for species delineation (Kim et al., 2014). Recent years, the ANI values of 95 to 96% is widely accepted as the threshold for species delineation, which was recognized as equal to the 16S rDNA gene sequence similarity of 98.65% (Kim et al., 2014; Richter and Rossello-Mora, 2009). The inter-clade ANI values together with the phylogenetic analyses confirmed the presence of three previously described *Fenollaria* species (i.e., *F. massiliensis* in clade A, *F. sporofastidiosus* in clade C and *F. timonensis* in clade D), In addition, the identification of a novel monophyletic lineage (clade B) suggested the presence a putative novel species within the genus *Fenollaria*, whose formal taxonomic characterization required further evidence of representative strains isolation, detailed phenotypic description and genomic evidence as well as full-length 16S rRNA gene sequences.

**Figure 3:**
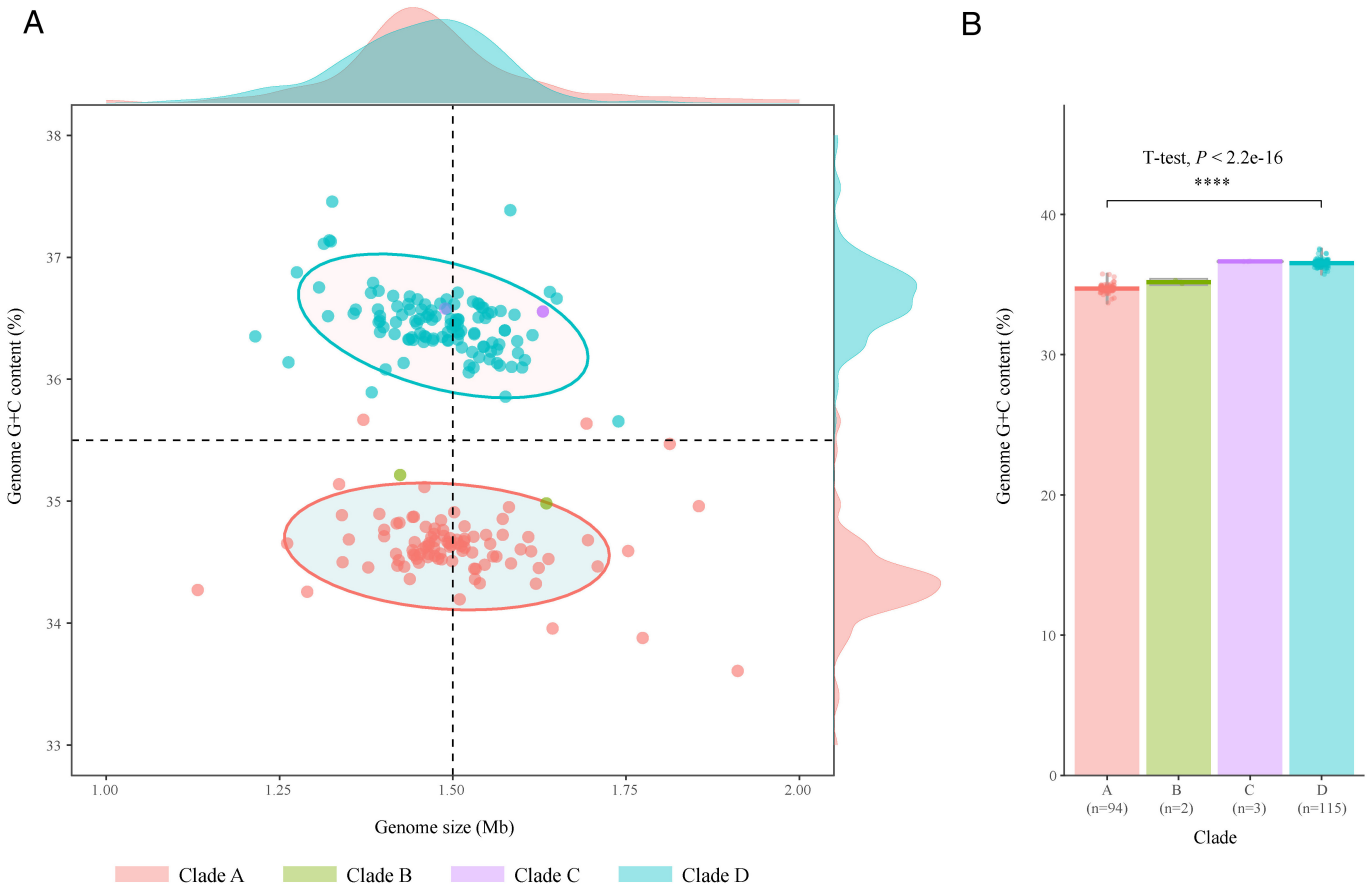
Genomic features distribution of *Fenollaria*. **(A)** Distribution of genome size along with genome G + C content. **(B)** Comparison of genome G + C content between clades.

The genomic differences between clades were also evaluated using two representative genomic features: genome size and G + C content. No significant differences in genome size were observed among clades, whereas the G + C content differed significantly (Figure 3A). Genomes in clade A exhibited a significantly lower G+C content of 34.60% ± 0.21% than those of 36.44% ± 0.18% in clade D (Figures 3A, B). In contrast, genomes in clade B showed a similar G + C content to those in clade A, while genomes in clade C showed a similar G + C content to those in clade D.

### 3.2 Pan-genome determination

**Figure 4:**
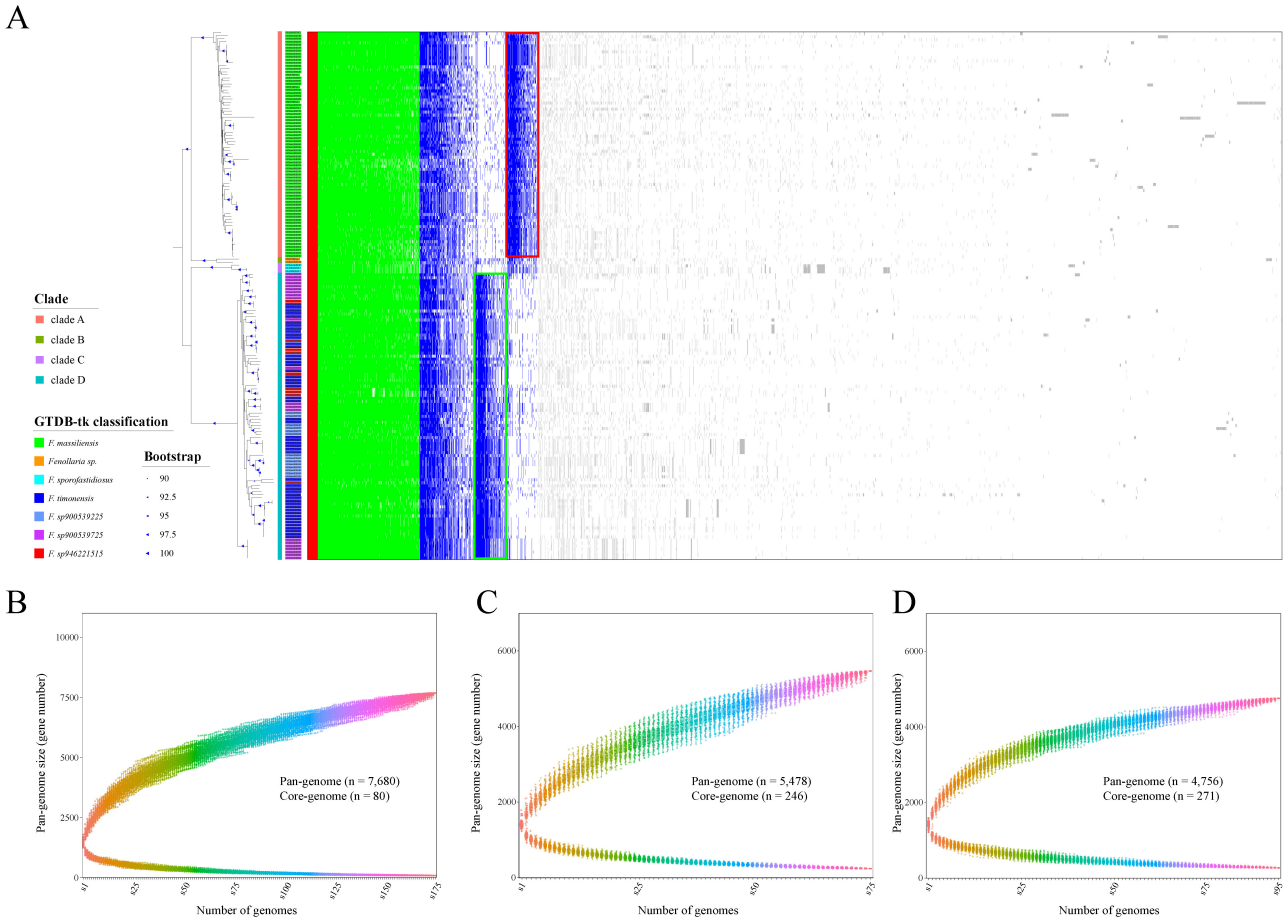
Pan-genome analysis of *Fenollaria*. **(A)** Heatmap of the gene presence/absence matrix mapped onto the phylogenomic tree. The hard-core, soft-core, shell and cloud-genes were marked in red, green, blue and gray on the heatmap, respectively. Genes specify enriched in clade A and clade B were highlighted with red and green boxes, respectively. **(B)** Gene accumulation curves for pan- and core-genomes of *Fenollaria*. **(C)** Pan- and core-genomes accumulation curves for clade A. **(D)** Pan- and core-genomes accumulation curves for clade D.

To further assess genomic conservation across species within the genus *Fenollaria*, a comparative pan-genome analysis was performed, categorizing genes into hard-core, soft-core, shell and cloud components. A pan-genome comprising of 7,680 genes clusters was constructed for the genus *Fenollaria* based on 175 genomes (Figures 4A, B). Only 80 genes clusters in the pan-genome were consistently present in all genomes (designated as hard-core genes), representing essential genes that encode fundamental cellular functions. The soft-core genome of genus *Fenollaria* consisted of 802 genes clusters, which contribute to species-specific adaptations (Nikolaidis et al., 2022; Goyal, 2018), including virulence factors, stress responses, and metabolic flexibility. In addition, 910 shell-genes were identified, which are typically involved in niche adaptation, environmental interactions, and host specificity (Gushgari-Doyle et al., 2022). Remarkably, a large number of cloud genes (*n* = 5,888) were observed, these genes were present in only a few strains and often acquired through horizontal gene transfer (Smith and Andam, 2021; Dmitrijeva et al., 2024), typically contributing to strain-specific traits such as antibiotic resistance, pathogenicity islands, or unique metabolic pathways. Overall, the core-genome was relatively small, leaving 88.52% of the total gene families classified as accessory genes. The pan-genome of *Fenollaria* remained open, as the number of genes clusters continued to increase with the addition of new genome (Figure 4B). This observation was further supported by Heaps' law analysis, which yielded a positive exponent (γ = 0.341), indicating an open pan-genome. Additionally, the pan-genome sizes of genomes in clade A and clade D were 5,478 and 4,756 (Figures 4C, D), both with similar pan-genome accumulation curves to genus *Fenollaria*.

Based on the distribution of gene presence/absence patterns across the phylogenomic tree, numerous clade-specific genes were observed (Figure 4A). As shown in the heatmap of gene presence/absence matrix, these clade-specific genes identified in clade A and D were classified as shell-genes. Using Scoary, a total of 182 clade-specific genes were identified with a Benjamini–Hochberg adjusted *P*-value ≤ 0.05, sensitivity > 0.95 and specificity > 0.95, including 18 genes in clade A, 26 genes for clade D and remained 138 genes for clade C. No clade-specific genes were identified for Clade B.

### 3.3 Distribution of functional categories

**Figure 5:**
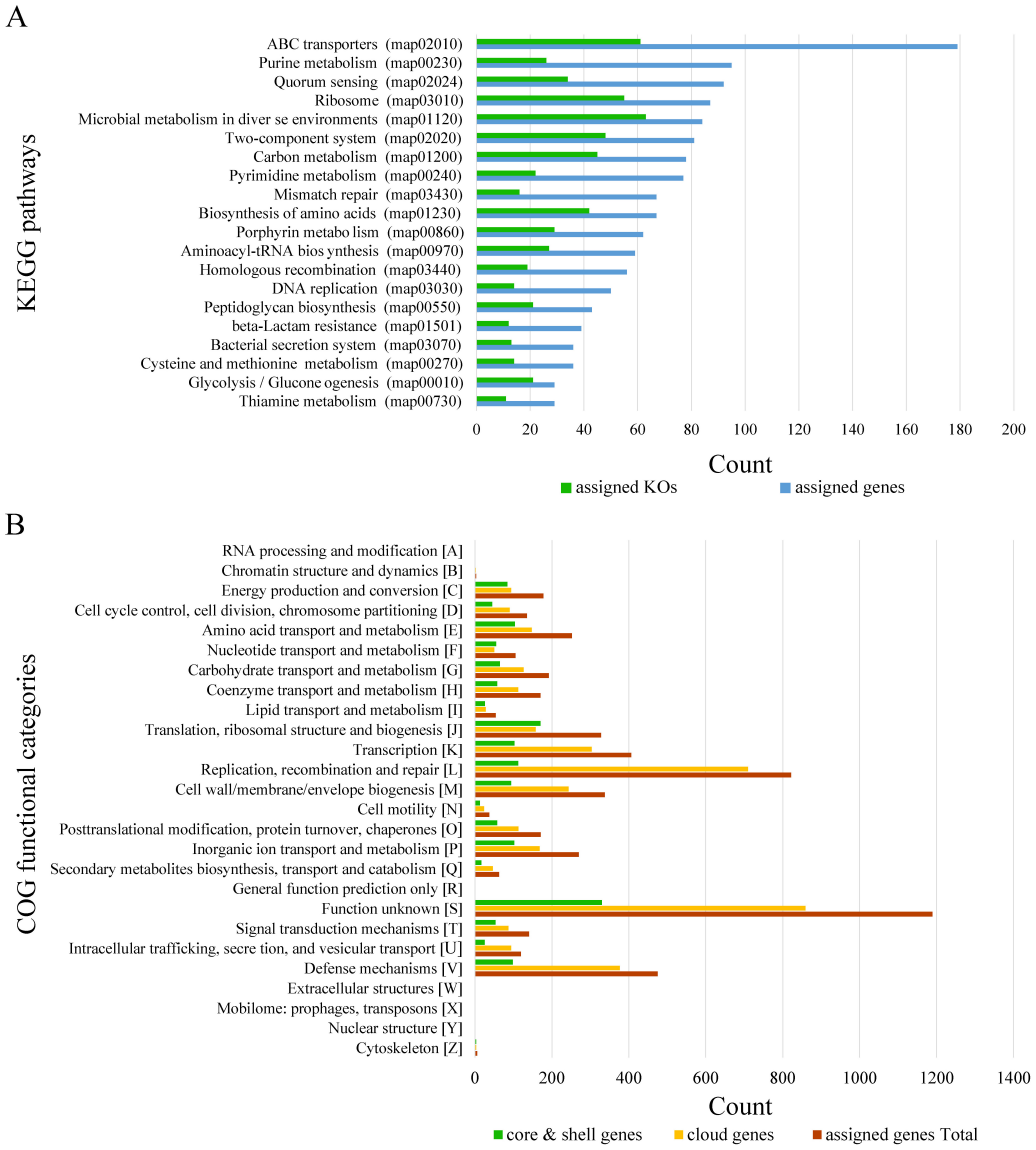
Functional genes distribution of *Fenollaria*. **(A)** Distribution of protein coding genes of *Fenollaria* pan-genome across KEGG pathways. The top 20 pathways were displayed. **(B)** Distribution of protein coding genes of *Fenollaria* pan-genome across COG functional categories.

The functional genes distribution of *Fenollaria* genomes was explored by assigning genes to KEGG pathways and COG categories. The number of genes and KO identifiers assigned to KEGG pathways were represented with bar graphs (Figure 5A). The ABC transporters, purine metabolism and quorum sensing pathways contained the largest numbers of annotated genes. In contrast, the ABC transporters, microbial metabolism in diverse environments and ribosome pathways contained the largest numbers of unique KO identifiers. The numbers of *Fenollaria* pan-genome genes assigned to each COG functional category, together with those of the core and shell genes and cloud genes were also represented by bar graphs (Figure 5B). Beside the function unknown [S] category, replication, recombination and repair [L] represented the largest functional category in the pan-genome. Genes involved in fundamental nutritional metabolism, including carbohydrate transport and metabolism [G], amino acid transport and metabolism [E], and energy production and conversion [C], were widely represented, indicating no obvious deficiencies in essential metabolic functions. Compared with the core and shell genes, the cloud genes were enriched in categories of replication, recombination and repair [L] and defense mechanisms [V].

**Figure 6:**
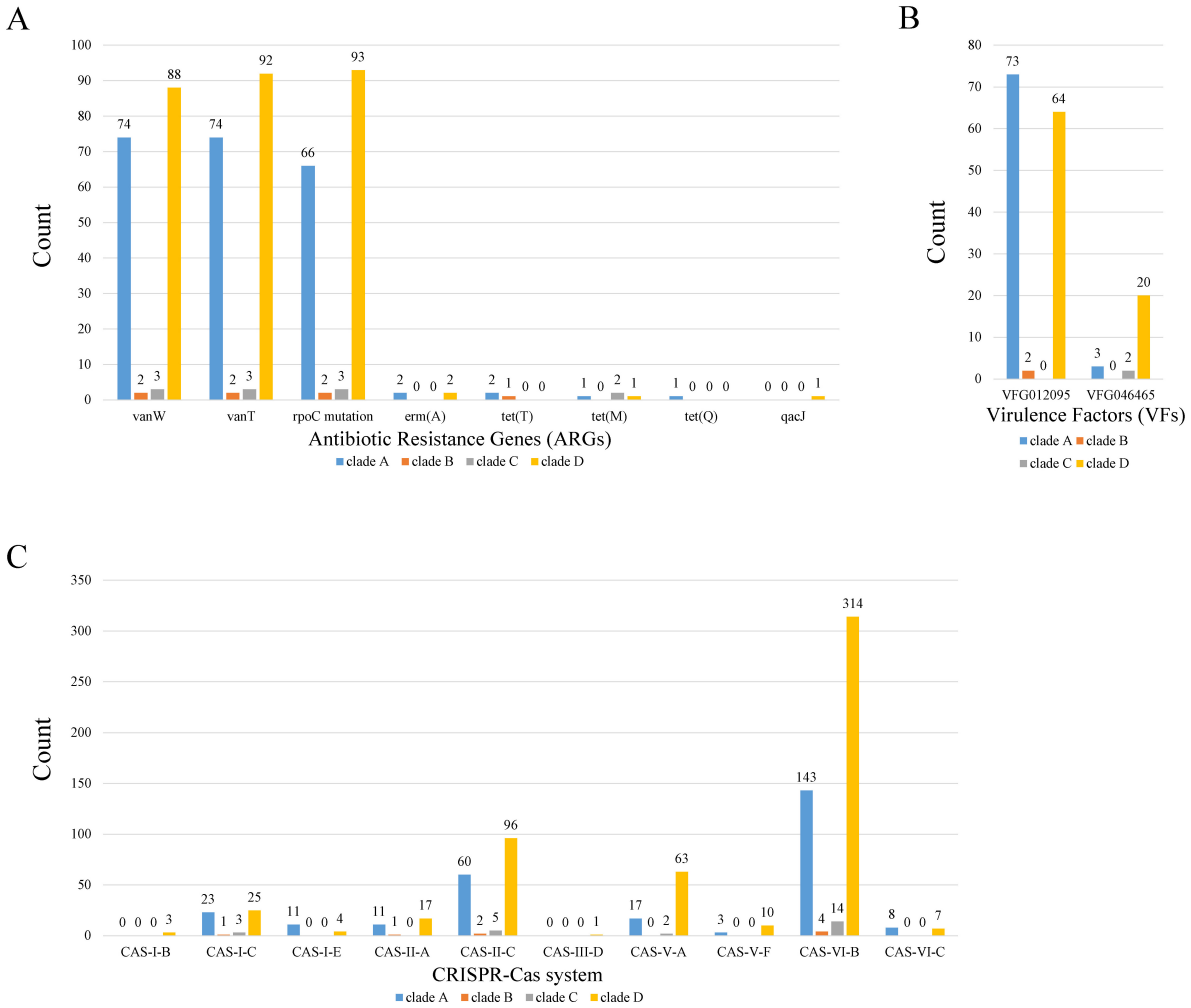
Functional characterization of *Fenollaria* genomes. **(A)** Antibiotic resistance genes (ARGs) identified in *Fenollaria* genomes. **(B)** Virulence factors (VFs) identified in *Fenollaria* genomes. **(C**) CRISPR-Cas systems identified in *Fenollaria* genomes.

Furthermore, genome-wide distributions of ARGs, VFs and CRISPR-Cas systems of each *Fenollaria* genome were conducted for deeper functional characterization. A total of eight ARGs were identified across the *Fenollaria* genomes (Figure 6A). Among them, the *vanT* gene, *vanW* gene and *rpoC* mutation were the prevalent ARGs, occurring in 171 (98%), 167 (95%), and 164 (94%) of the 175 genomes, respectively. These ARGs were associated with antibiotic target alteration and were predicted to confer resistance to glycopeptide antibiotics (vancomycin and teicoplanin) and rifamycins (rifampicin and rifabutin).

Two colonization-associated VF homologs were identified in *Fenollaria* genomes, i.e., VFG012095 and VFG046465 (Figure 6B). Both homologs belonged to the Colonization_VF category, which suggested their potential roles in host adaptation and ecological persistence. The VFG012095 homologs were detected in 139 of 175 genomes (79.4%). Its distribution varied among clades, being present in 73 of 75 genomes (97%) in clade A, two of two genomes (100%) in clade B, absent from clade C (0 of 3 genomes) and detected in 64 of 95 genomes (67%) in clade D. The VFG046465 homologs showed a more biased distribution, being detected in 25 of 175 genomes (14%). They were present in three of 75 genomes (4%) in clade A, absent from clade B (0 of 2 genomes), detected in two of three genomes (67%) in clade C, and identified in 20 of 95 genomes (21%) in clade D.

A total of ten CRISPR-Cas types were identified across *Fenollaria* genomes (Figure 6C). The CAS-VI-B CRISPR-Cas system was identified in all 175 genomes, represented the most prevalent CRISPR-Cas subtype. The type CAS-II-C CRISPR-Cas system was identified in 150 of 175 genomes (85.71%). They were present in 58 of 75 genomes (77.33%) in clade A, detected in all genomes from clades B and C, and identified in 87 of 95 genomes (91.58%) in clade D.

## 4 Discussion

The genus *Fenollaria* had attracted increasing attention due to its emerging association with PCa (Hurst et al., 2022), CRC (Cai et al., 2025) and several other human diseases. However, these associations were primarily inferred from species abundance difference between individuals. The genomic landscape of *Fenollaria* remains largely unexplored. A large-scale comparative genomics analysis was constructed to infer intra-genus phylogenetic relationships and distributions of genomic characterizations of genus *Fenollaria*.

To data, only five isolate genomes, represented three species of *Fenollaria*, have been determined, and research on each species is relatively independent. The *F. massiliensis* was initially isolated from an osteoarticular sample with non-contiguous finished genome sequenced (Pagnier et al., 2014). Boiten et al. (2018) mostly encountered this species in clinical samples from the groin region, and proposed 16S rDNA gene sequencing for insight the clinical relevance of *F. massiliensis*, even the genome of the representative strain has been sequenced. France et al. (2022) reported the announcements of the other *F. massiliensis* genome, still leaving genomes largely unexplored. *F. timonensis* was designed as based on 16S rDNA sequence identity of 97.4% against *F. massiliensis*, and no comparative genomics analysis was constructed between the two species (Durand et al., 2017; Lo et al., 2020). Hurst et al. (2022) established association of *Fenollaria* sp. nov. and unfavorable clinical outcomes in PCa patient and constructed the whole genome of *F. sporofastidiosus*, however its genomic characteristics have not been systematically compared with those of other described *Fenollaria* species. Here, four mono-clades with clear genomic divergence were proposed. Beside three of these clades corresponded to previously described species, respectively, a fourth clade was proposed, supported by two adjacent MAGs. Biased distributions of ANI and AAI values were observed and the genomes in clade A exhibited a significantly lower G + C content than those in clade D.

Beyond taxonomic resolution, functional characterization based on large-scale genomic datasets is also essential for the genomic landscape of *Fenollaria*. Metabolic pathway reconstruction and investigations of ARGs, VFs and CRISPR-Cas system provided fundamental support for laboratory culture and ecological adaptation mechanism exploration. No essential metabolic deficiency was observed in *Fenollaria*. Resistance to glycopeptide antibiotics (vancomycin and teicoplanin) and rifamycins (rifampicin and rifabutin) of *Fenollaria* was revealed. The existence of colonization-associated VFs suggested their potential roles in host adaptation and ecological persistence. The CAS-VI-B and CAS-II-C CRISPR-Cas systems in *Fenollaria* genomes suggested adaptive immunity strategy by targeting foreign genetic material. These results partially filled the gap in previous genomic analysis of *Fenollaria*.

Although a large number of genomes were included and multiple genomic features were characterized, several limitations still remained. There were only two and three genomes in clade B and C, respectively. The limited number of genomes available for clades B and C (two and three genomes, respectively) may result in an incomplete investigation of the genomic diversity and functional characteristics within these lineages. In particular, no representative isolate genome was available for clade C, and full-length 16S rRNA gene sequences were lacking. limiting further taxonomic resolution. Therefore, the proposed taxonomic assignments for species in clade C required additional evidence, including representative strains isolation, detailed phenotypic description and genomic evidence as well as full-length 16S rRNA gene sequences. The taxonomic classification of genomes within clade D was inconclusive, as at least four species were identified within this clade, but none of the species formed mono-subclade. Overall, the functional characteristics were primarily inferred from genome-based bioinformatic analyses and further experimental validation were require. The mechanisms underlying the associations between *Fenollaria* and human diseases, such as PCa and CRC have not been elucidated. Laboratory cultivation, transcriptomics and metabolomics analysis might be required to address these issues in subsequent studies.

## Data Availability

The datasets presented in this study can be found in online repositories. The names of the repository/repositories and accession number(s) can be found in the article/Supplementary material.
